# Six Recommendations for Accelerating Uptake of National Food Security Screening in Primary Care Settings

**DOI:** 10.1007/s11606-021-07137-1

**Published:** 2021-09-24

**Authors:** Sabira Taher, Stephen D. Persell, Namratha R. Kandula

**Affiliations:** 1 Department of Preventive Medicine, Feinberg School of Medicine, Northwestern University, 680 N Lake Shore Drive Suite 1400, Chicago, IL 60611 USA; 2 Division of General Internal Medicine and Geriatrics, Department of Medicine, Feinberg School of Medicine, Northwestern University, Chicago, IL USA; 3 Center for Primary Care Innovation, Institute for Public Health and Medicine, Feinberg School of Medicine, Northwestern University, Chicago, IL USA; 4 Center for Center for Community Health, Institute for Public Health and Medicine, Feinberg School of Medicine, Northwestern University, Chicago, IL USA

## INTRODUCTION

Food insecurity is the limited access to nutritious food that prevents people from living active and healthy lives. Importantly, it is associated with nutritional deficiency, obesity, and chronic disease health disparities across the USA.^1^ For the past decade, roughly one in eight US households was food insecure, with lower-income, minority households experiencing a greater burden.^2^ Over the past 18 months, the COVID-19 pandemic has created an economic crisis for many individuals and the demand for food assistance has nearly doubled.^2^ Food assistance comes in many forms. Federally funded programs include the Supplemental Nutrition Assistance Program (SNAP), the Special Supplemental Program for Women, Infants and Children (WIC), and school meal programs. These programs serve one in four Americans and actively work with non-profit and public organizations, such as food pantries and public schools to make healthy and nutritious food more accessible and affordable for children and low-income families.^1^

A multi-sector response that integrates clinical care and community food assistance services is a powerful lever to ameliorate this growing crisis. Studies have demonstrated effective collaborations between primary care and community organizations to assist with chronic disease management through coordinated care and increased access to unmet social needs.^3,4^ Evidence also points to multi-sector interventions to address food insecurity in response to national organizations calling for clinical food security screening in primary care practice.^1,3,5^

## RECOMMENDATIONS FOR CLINICAL FOOD SECURITY SCREENING

Government and professional organizations, such as the American Academy of Family Physicians and the Centers for Medicaid and Medicare Services, recognize primary care settings as the foundation for integrating clinical care and community-based services.^6^ To that end, they recommend using the two-question Hunger Vital Signs tool within EHR systems to automate the process of identifying patients already experiencing some food insecurity and generate referrals to SNAP and local food assistance resources.^3^

### Clinical Food Security Screening in Practice

In practice, uptake of clinical screening recommendations proves challenging. A recent study showed that only 16% of primary care practices screened for social determinants of health (SDOH), including food security, even though clinicians agreed that they would screen for it more often if they were better prepared to address food insecurity (9,10). There is some evidence that barriers include clinician lack of time and knowledge about how to ask patients about food insecurity and, most importantly, how to refer and link patients to food assistance resources..^1,7^

In response, health systems have leveraged the strength of community food organizations to increase clinician capacity to address food insecurity. Mutually beneficial partnerships between health systems and neighborhood food pantries, farmers markets, and/or community gardens have provided access to free and lower-cost healthy food through produce prescription programs, nutrition education, and enrollment in SNAP benefits. In many cases, clinician training was also provided, which was meant to prepare providers with the appropriate skills necessary to ask and counsel patients about food insecurity.^1,3,5^ Yet, the frequency that clinicians utilized available training resources has not been linked to increased food security screening practices and utilization. Impact of trainings should be measured in future research.

While this type of multisector approach demonstrates immense potential for large health systems, clinical-community partnerships require a considerable investment of time, staff, and financial capital to establish and sustain.^1,4^ Therefore, this approach may not be feasible for resource challenged healthcare settings, including small practices and community clinics. Recognizing the need to prioritize food insecurity in diverse primary care settings, we make pragmatic recommendations that are more feasible.

#### Three Recommendations for Health Systems

1. *Tailor food security screening processes to fit clinical workflows*: National recommendations lack clarity about how and when screening and referral processes should be integrated into the clinical workflow. Evidence points to specific clinic-level adaptations that enabled uptake of context-specific screening and referral practices at various patient touch points.^1,3^ For example, health systems may be able to more reliably, and efficiently screen patients if they use more than one screening modality such as a self-administered pre-visit screening tool delivered through the online patient portal, and distribution of written screening questionnaires in person for patients who did not complete it before the visit.^1^
2. *Improve clinician awareness about food insecurity and local resources*: Census-level and EHR data can be used to demonstrate the patterns of food insecurity, and overlapping social needs, such as affordable housing, low wages, and unemployment. When food insecurity data were presented at regular staff meetings, one study showed an increase in clinician knowledge and motivation to address food insecurity.^1,7^ Clinicians should be educated about location-specific resources that can fulfill these basic needs.
3. *Generate a local resource referral list*: Collaborate with staff already tapped into the community (e.g., social workers, patient navigators, and community health educators), and map out location-specific food assistance resources. One study made a resource list accessible for both clinicians and patients by providing handouts of the list in the patient waiting room, exam rooms, nurse’s station, break room, and at patient summary report.^1,3^ Using online resources that provide community asset mapping and facilitate referrals, such as the community referral platform NowPow, can be customized and automated within EHR systems to meet the needs of both patients and care teams.^1^

#### Three Recommendations for Clinicians

1. *Reduce patient discomfort and build trust during screening*: Clinicians should aim to reduce patient discomfort by conducting screening in a private setting.^1^ Preface the conversation with, “I ask all of my patients about access to nutritious food because it’s such an important part of managing one’s health.” Evidence points to statements like this that can generate patient trust and articulate the goal of offering support.^1,4,7^
2. *Ask about SNAP and WIC*: One effective way to increase use of SNAP and WIC benefits is to directly ask Medicaid beneficiaries if they are enrolled in these programs and refer them to appropriate resources.^4^
3. *Advocate for local resources*: Non-profit organizations have very limited resources for effective dissemination of information, which contributes to chronic underuse of services.^1^ Primary care clinicians can use their influential voice and status as trusted members of the community to increase visibility of local food resources. Research shows that when primary care clinicians are responsible for making referrals to specific services, patient use of those programs increased.^6^

## CONCLUSION

Translating evidence into practice is often difficult for healthcare settings—especially resource-challenged clinics that serve populations burdened by social and economic inequity. Keeping this in mind, the suggested adaptations of food security screening practices can increase feasibility, uptake, efficiency, and equity across US primary care settings.
